# Diversity of transgene integration and gene-editing events in wheat (*Triticum aestivum* L.) transgenic plants generated using *Agrobacterium*-mediated transformation

**DOI:** 10.3389/fgeed.2023.1265103

**Published:** 2023-12-19

**Authors:** Louie Cris Lopos, Natalia V. Bykova, Janeen Robinson, Susan Brown, Kerry Ward, Andriy Bilichak

**Affiliations:** Agriculture and Agri-Food Canada, Morden Research and Development Centre, Morden, MB, Canada

**Keywords:** wheat, *Triticum aestivum* L., *Agrobacterium*-mediated transformation, gene editing, CRISPR/Cas9, epigenetics

## Abstract

Improvement in agronomic traits in crops through gene editing (GE) relies on efficient transformation protocols for delivering the CRISPR/Cas9-coded transgenes. Recently, a few embryogenesis-related genes have been described, the co-delivery of which significantly increases the transformation efficiency with reduced genotype-dependency. Here, we characterized the transgenic and GE events in wheat (cv. Fielder) when transformed with *GROWTH-REGULATING FACTOR 4* (*GRF4*) and its cofactor *GRF-INTERACTING FACTOR 1* (*GIF1*) chimeric gene. Transformation efficiency in our experiments ranged from 22% to 68%, and the editing events were faithfully propagated into the following generation. Both low- and high-copy-number integration events were recovered in the T_0_ population with various levels of integrity of the left and right T-DNA borders. We also generated a population of wheat plants with 10 different gRNAs targeting 30 loci in the genome. A comparison of the epigenetic profiles at the target sites and editing efficiency revealed a significant positive correlation between chromatin accessibility and mutagenesis rate. Overall, the preliminary screening of transgene quality and GE events in the T_0_ population of plants regenerated through the co-delivery of *GRF–GIF* can allow for the propagation of the best candidates for further phenotypic analysis.

## 1 Introduction

Bread wheat (*Triticum aestivum* L.) is one of the staple crops providing more than 20% of calories to the population worldwide (Walkowiak et al., 2020). The Green Revolution in the 1960s led to an increase in grain productivity in wheat owing to the introduction of the semi-dwarf alleles *Rht1* (*Rht-B1b*) and *Rht2* (*Rht-D1b*) (Davière and Achard, 2016). The phenotype of both alleles is caused by the gain-of-function (GoF) mutation of the DELLA-coding genes (*Rht1* and *Rht2* are homeologous genes on chromosomes 4B and 4D, respectively), leading to overaccumulation of the protein that restrains plant height. DELLA GoF mutation also causes several negative pleiotropic effects, including shorter coleoptile length, reduced seedling vigor, grain weight, lower nitrogen-use efficiency, and tolerance to drought compared to tall varieties (Rebetzke et al., 2007). Therefore, a further increase in wheat yield should be focused on eliminating the negative pleiotropic effects caused by the semi-dwarf genes and creating a new genetic variation with beneficial agronomic traits. The polyploid (allohexaploid, AABBDD) and complex nature of the wheat genome (16 Gb, 85% of repetitive elements) (Walkowiak et al., 2020) make it challenging to rapidly achieve improvement in the agronomic traits using natural or induced random genetic variation. GE, through the CRISPR/Cas technology, offers an alternative approach for generating the induced variation at the target sites in the genome’s regulatory or coding regions. Additionally, targeted generation of the double-strand breaks (DSBs) allows, for example, induced recombination at the predefined positions (Chen et al., 2022), introgression of specific loci from the close relatives or other cultivars, and elimination of linkage drag (Lazar et al., 2020). Until recently, the stable wheat transformation was relatively challenging due to its high variability and genotype-dependency, resulting in low transformation efficiency (1%–2%). The delivery of growth-regulating and regeneration-related genes such as *TaWox5* (Wang et al., 2022), maize *Zm-Baby Boom* (*ZmBbm*) and *Zm-Wuschel2* (*ZmWus2*) (Johnson et al., 2023), and *GROWTH-REGULATING FACTOR 4* (*GRF4*) and its cofactor *GRF-INTERACTING FACTOR 1* (*GIF1*) *GRF–GIF* (Debernardi et al., 2020) results in a significant boost in transformation efficiency ranging from 58% to 75%. Although higher transformation efficiency allows for reproducible generation of transgenic plants carrying the CRISPR/Cas9 constructs in different experiments, editing efficiency at different loci still varies significantly. Numerous methods have been created to forecast the effectiveness and mutation results by relying exclusively on CRISPR target sequences (Allen et al., 2018; Concordet and Haeussler, 2018; Xiang et al., 2021). Nevertheless, the reliability of these tools, which are primarily based on human cell data, frequently differs and exhibits limited applicability when extended to plants (Naim et al., 2020). This finding implies that factors other than the sequence itself might impact CRISPR/Cas9 mutagenesis. Indeed, the recent report provided direct evidence that DNA methylation and chromatin characteristics can result in substantial fluctuations in mutagenesis efficiency, with differences of up to 250 times observed (Weiss et al., 2022). The editing rate at distinct loci in the *Arabidopsis thaliana* genome carrying the same gRNA-binding sequences did not depend on the sequence itself, with lower efficiencies of mutagenesis predominantly linked to repressive heterochromatic features. The repressive impact could be mitigated by significantly reducing DNA methylation levels at the targeted CRISPR sites. Additionally, specific chromatin attributes, such as H3K4me1, H3.3, and H3.1, were found to be associated with notable variations in the patterns of CRISPR/Cas9 mutations facilitated by the non-homologous end-joining (NHEJ) repair pathway.

The complex wheat genome exhibits a multi-tiered spatial organization encompassing three levels (Concia et al., 2020a). First, the genome is structured into distinct territories, where specific regions occupy defined spatial locations within the nucleus. Second, a clear demarcation between facultative (modifiable) and constitutive (invariant) heterochromatin forms a distinct separation. Lastly, the organization of RNA polymerase II around transcription factories contributes to the overall spatial arrangement of the genome, indicating a functional relationship between transcriptional activity and genomic organization. Such complexity undoubtedly introduces differences in GE rates among different gRNAs, simply by restricting the access of the Cas9/gRNA complex to the target regions. In this study, we took advantage of the extensive collection of high-resolution epigenomic data already available for wheat to investigate the influence of chromatin characteristics on CRISPR/Cas9 mutagenesis. We employed stable transgenic wheat plants as our experimental model generated by *Agrobacterium*-mediated transformation and *GRF–GIF* chimera. Here, we report on the quality of transgenic events obtained through this method as well as demonstrate the correlation between the chromatin accessibility (as measured through Assay for Transposase-Accessible Chromatin-sequencing, ATAC-Seq) and GE efficiency for 10 gRNAs tested in our study.

## 2 Materials and methods

### 2.1 Plant cultivation

Donor and transgenic plants (cv. Fielder) were grown in Sunshine^®^ Mix #4 Aggregate Plus potting mix with Osmocote 14-14-14 slow-release fertilizer in growth cabinets at 20°C day and 15°C night temperatures, 16/8 h day/night regime, and 600 µmol/m^2^s light intensity provided by fluorescent tubes. Humidity was maintained at 70%. Extra care was taken to keep donor plants healthy and reduce the risk of spreading pathogens. Powdery mildew was controlled through weekly foliar application of the plant health promoter Foliar Supreme S720 (2 mL/L, OMEX Agriculture Inc., Canada) until the heading stage. The thrip population was controlled through the weekly application of the diatomaceous earth to the heads and leaves of the donor plants.

### 2.2 *Agrobacterium*-mediated wheat transformation

The wheat transformation was done as described in Hayta et al. (2021). In brief, following seed sterilization, immature embryos were collected at the early milk stage (∼10–13 days post-anthesis, 1–2 mm in diameter) into 1.7-mL microcentrifuge tubes containing 1 mL of wheat inoculation medium (WIM—0.44 g/L Murashige and Skoog, 10 g/L glucose, and 0.5 g/L 2-(N-morpholino) ethanesulfonic acid (MES)) with 0.05% Silwet L-77 (∼100 embryos/tube). The WIM was replaced with the fresh media, and embryos were centrifuged for 10 min at 14,000 rpm at 4°C. The transformation was performed using the hypervirulent *Agrobacterium tumefaciens* strain AGL1 carrying the pSoup helper plasmid and the JD633 plasmid containing the cloned gRNA seed sequences. In some cases, the *Agrobacterium* cultures with two different gRNAs were mixed in equal volumes immediately before embryo inoculation for co-transformation experiments. WIM was removed, and 1 mL of *Agrobacterium* solution (WIM supplemented with 100 μM acetosyringone, OD_600_ = 0.5) was added; tubes were inverted repeatedly for 30 s and incubated for at least 20 min. The suspension was removed with the pipette, and 25–30 embryos were transferred to fresh 90-mm-diameter plates, scutellum side up, with a wheat co-cultivation medium (WIM supplemented with 100 μM acetosyringone, 5 μM AgNO_3_, 1.25 mg/L CuSO_4_·5H_2_O, and 8 g/L agarose). Petri plates were sealed and incubated in the dark at 24°C for 3 days, followed by excision of the embryogenic axes. The embryos, scutellum side up, were transferred to fresh wheat callus induction medium plates (WCI—4.3 g/l MS, 30 g/L maltose, 1.0 g/L casein hydrolysate, 10 mL 100× vitamin stock, 2 mg/L picloram, 0.5 mg/L 2,4-D, 1.25 mg/L CuSO_4_·5H_2_O, 160 mg/L timentin, and 5 g/L agarose) and incubated at 24°C in the dark for 5 days. Following the resting period, the embryos were transferred to fresh WCI plates supplemented with 15 mg/L hygromycin and kept at 24°C in the dark for 2 weeks. Eventually, the calli were split into clumps of ∼4 mm^2^ and transferred to fresh selection plates (WCI plates) but with 30 mg/L hygromycin. The plates were moved back to 24°C in the dark for 2 weeks, then transferred to a lit culture chamber (100 μmol/m^2^/s) at the same temperature with a 16-h photoperiod, and covered with a single-layer paper towel for a week. The transformed calli started to turn green and produce small shoots during this period. The calli were transferred a final time to a wheat regeneration medium (WRM—4.3 g/l MS, 30 g/L maltose, 1.0 g/L casein hydrolysate, 10 mL vitamin stock, 1.0 mg/L zeatin, 1.25 mg/L CuSO_4_·5H_2_O, 3.5 g/L phytagel, 160 mg/L timentin, and 20 mg/L hygromycin) and kept under fluorescent lights (100 μmol/m^2^/s) at 24°C with a 16-h photoperiod. The regenerated shoots with prominent roots were transferred to magenta jars containing the WCI medium without growth regulators and supplemented with 15 mg/L hygromycin. The well-developed shoots with strong root systems were eventually transferred to soil and covered with a plastic dome for acclimatization.

### 2.3 Cloning of gRNAs and PCR confirmation of transgenic plants

The wheat transformation was done with the JD633 plasmid containing the TaCas9 expression cassette together with TaU6:gRNA and the *GRF4–GIF1* embryogenesis chimeric gene (Debernardi et al., 2020). The plasmid was a gift from Jorge Dubcovsky (Addgene plasmid # 160393; http://n2t.net/addgene:160393; RRID: Addgene_160393). Complementary oligos (Supplementary Table S6) were annealed (2 µM each in 50 µL of water), and 1 µL of the annealed product was mixed with 100 ng of the *Aar*I-digested vector, 1x T4 DNA ligase buffer, and 400 U of T4 DNA ligase. The ligation mix was incubated for 1 h at room temperature and transformed into *E. coli* commercial chemical competent cells. Cloned seeding sequences were verified by the Sanger sequencing method, and the plasmids were transformed into the *A*. *tumefaciens* strain AGL1 (pSoup) using a chemical transformation protocol (An et al., 1988). gDNA was isolated from leaves of the soil-acclimated putative transgenic plants, and the presence of the transgene was confirmed using PCR with HYG-specific primers (HYG-F and HYG-R, Supplementary Table S6). Segregation analysis for the T_1_ plants was done using the same HYG-specific primers and with up to 10 plants per T_0_ transgenic line.

### 2.4 Evaluation of GE at the target site using cleaved amplified polymorphic sequences, qPCR assays, and sequencing confirmation

The editing efficiency at the target sites was assessed using either the cleaved amplified polymorphic sequence (CAPS) assay, as described previously (Xu et al., 2022), or the qPCR method. In the CAPS assay, the gRNA#10 target regions were amplified using sub-genome-specific or universal primers (Supplementary Table S6) and digested with *Sma*I. The digested products were separated on 1% agarose gel, and the editing efficiency was calculated as a percentage of undigested band intensity measured using Image Lab software (Bio-Rad, United States).

A multiplexed probe-based qPCR method was used to assess the editing efficiency for gRNAs tested in this study. The primers were designed to amplify the editing region with the 5′-FAM (6-fluorescein)-labeled probe annealing to the cleavage sites of the gRNAs for three sub-genomes (A, B, and D, Supplementary Table S6). The *PINb* gene (*puroindoline-b*, two copies per hexaploid genome) was used as a reference (Collier et al., 2017). The reference gene probe was 5ʹ-HEX (hexachlorofluorescein)-labeled, and both gRNA and *PINb* gene probes contained ZEN and Iowa Black Hole Quencher 1 (Integrated DNA Technologies, Coralville, IA, United States). The 1x Luna^®^ Universal Probe qPCR Master Mix (cat. #M3004S; New England Biolabs, United States), 500 nM of each primer pair (for the endogenous reference gene and the transgene), and 250 nM each of gRNA and reference probes were mixed in the total volume of 20 μL. qPCR was run on the CFX96 Real-Time PCR Detection System (Bio-Rad) with 50 ng/sample of gDNA. PCR conditions consisted of one cycle of initial denaturation at 95°C (60 s), followed by 45 cycles of denaturation at 95°C (15 s) and extension at 60°C (30 s). Reactions were run in triplicate, and average ΔΔCt was calculated for transgenic and wild-type control (WT, cv. Fielder) samples for corresponding gRNAs using CFX Manager, v. 3.1 (Bio-Rad). The GE rates were calculated through the normalization of ΔΔCt values of transgenic plants to WT control (ΔΔCt_norm_), and the 1/ΔΔCt_norm_ ratios were used for analysis.

Target regions for three selected gRNAs (gRNAs #2, 4, and 6) were PCR-amplified with either sub-genome-specific or universal primers (Supplementary Table S6) using gDNA of the T_1_ plants that demonstrated edits in the qPCR assay. Fragments were analyzed on the gel and cloned into the pJET1.2 plasmid (cat. #K1231, CloneJET PCR Cloning Kit, Thermo Fisher Scientific). Sequencing was done using Primordium Labs (United States) and analyzed using Geneious Prime software.

### 2.5 Estimation of the transgene copy number

The T-DNA copy number in T_0_ plants and their progeny was measured using digital droplet PCR (ddPCR), as described by Collier et al. (2017), with some modifications. gDNA was isolated from young leaves using the method that was developed by Warner et al. (2002). The concentration was quantified using a Qubit fluorometer and the dsDNA quantification kit (cat. #Q32851), as described by the manufacturer (Thermo Fisher Scientific, Waltham, MA, United States). For every sample, 1 µg of gDNA was digested either for 1 or 12 h at 37°C using *EcoR*I-HF (cat. #R3101S) in 1x rCutSmart Buffer, followed by heat inactivation at 65°C for 20 min. The digested gDNA was purified using the DNA Clean-Up and Concentration Micro-Elute Kit (cat. #67200, Norgen Biotek Corp., ON, Canada) and quantified again using a Qubit fluorometer. Two different amounts of digested gDNA/reaction were tested and compared for the efficiency of transgene copy number estimation—350 ng (Collier et al., 2017) and 10 ng (Jouanin et al., 2020). The transgene-specific primers/probe mix was explicitly designed for TaCas9 (Supplementary Table S6), and normalization was done against the *PINb* gene. The transgene and reference gene probes were labeled with 5′-FAM (6-fluorescein) and 5ʹ-HEX (hexachlorofluorescein). All probes were double-quenched with ZEN and Iowa Black Hole Quencher 1 (Integrated DNA Technologies, Coralville, IA, United States). The ddPCR master mix for amplification contained 1x ddPCR Supermix for Probes (no dUTP; cat. # 186-3024; Bio-Rad Laboratories), 500 nM of each primer pair (for the endogenous reference gene and the transgene), and 250 nM of each probe. The total volume was adjusted to 20 µL with the ultrapure water. The droplets were produced following the manufacturer’s instructions using a droplet generator (Bio-Rad) and Bio-Rad Droplet Generation Oil (cat. # 186-3005). Droplets (40 µL) were transferred to a 96-well skirted Eppendorf PCR plate (cat. # 951020362), sealed with pierceable foil (cat. # 181-4040; Bio-Rad) and placed into the Bio-Rad thermocycler. Initial denaturation was done for 10 min at 95°C, followed by 40 cycles of 94°C (30 s) and 60°C (1 min) with a final step at 98°C for 10 min. A temperature ramp rate was set to 2°C/s, according to the manufacturer’s instructions. The samples were then transferred to a QX200 droplet reader (Bio-Rad). Data analysis was done using the Bio-Rad QuantaSoft Analysis Pro software (v1.0.596) with manual settings for threshold determination to distinguish positive and negative droplets.

### 2.6 Evaluation of the left and right T-DNA border integrity and transgene co-integration events

We used end-point PCR to estimate the intactness of the left and right border (LB and RB, respectively) integration events in the T_0_ plants. For RB, forward primer (RB-F, Supplementary Table S6) was used in combination with either RB-R1 or RB-R2 primers, one of which anneals at RB (RB-R2, L = 355 bp), whereas another one is 122-bp 5′-upstream from RB (RB-R1, L = 233 bp). The PCR reaction was run on 1% agarose gel, and the presence of the larger band was indicative of the intactness of RB. For LB, common reverse primer (LB-R) was mixed with either of the three forward primers annealing either at LB (LB-F1, L = 500 bp), 75-bp 3′-downstream (LB-F2, L = 425 bp), or 317-bp downstream (LB-3, L = 183 bp). The presence of the 500-bp PCR fragment indicated the integrity of LB.

In experiments where two *Agrobacterium* cultures carrying different gRNAs were mixed, co-integration of T-DNAs was assessed using PCR with the common primer for the JD633 vector backbone (JD633-F) and reverse primer specific to the gRNA seeding sequence (Supplementary Table S6). The PCR reactions were analyzed separately on 1% agarose gel for every T_0_ transgenic plant.

### 2.7 Gene expression analysis using ddPCR

Total RNA was isolated from leaves of transgenic T_1_ seedlings using a NucleoSpin RNA Plant Kit (cat. # 740949.50, Macherey-Nagel, Germany), according to the manufacturer’s instructions. cDNA was synthesized using a SuperScript IV First-Strand Synthesis System (cat. # 18091050, Thermo Fisher Scientific, United States), following the manufacturer’s protocol. For every ddPCR, 20 ng of cDNA/sample was used and combined with 1x ddPCR Supermix for Probes (no dUTP; cat. # 186-3024; Bio-Rad Laboratories), 500 nM of each primer pair (for the endogenous reference gene and the transgene), and 250 nM of each probe. Expression from the transgene cassette was measured using ddPCR (Bio-Rad, United States) with TaCas9 primers/probe (Supplementary Table S6). *TaGA3PD* (TraesCS7A02G313100, TraesCS7B02G213300, and TraesCS7D02G309500) was used as an endogenous reference (Garrido et al., 2020) (*TaGA3PD*, 5′-HEX-labeled, double-quenched with ZEN and Iowa Black Hole Quencher 1, Supplementary Table S6). ddPCR gene expression data were normalized as previously described (Coulter, 2018).

### 2.8 Characterization of chromatin features

The chromatin marks for the wheat (cv. Chinese Spring) were extracted from the following datasets—SRP126222 (H3K27me3, H3K36me3, H3K4me3, and H3K9ac), SRP133674 (DNA methylation) (Concia et al., 2020a), GSE133885 (ATAC-Seq), and GSM3929161 (RNA polymerase II ChIP-Seq) (Jia et al., 2021). The gRNA regions, including the three-nucleotide protospacer adjacent motif (PAM), were BLAST-searched using the WheatOmics 1.0 browser (http://wheatomics.sdau.edu.cn/), and nucleotide resolution data were extracted for corresponding chromatin marks. The average value across the respective gRNA target sequence plus PAM was calculated for three sub-genomes. The data set for every chromatin mark was normalized for comparison on a scale of 0–1, with 1 indicating the highest level of that feature, and used to construct the heatmap.

The ATAC-Seq coverage data for wheat chromosomes (IWGSC RefSeq v1.0 (International Wheat Genome Sequencing, 2018)) were plotted using the R package karyoploteR (Gel and Serra, 2017). Eventually, the gRNA and centromere positions were displayed as markers.

### 2.9 Statistical treatment of the data

Statistical significance between groups for correlation analysis was calculated using Pearson’s correlation test, with *p* < 0.05 considered significant.

## 3 Results

### 3.1 *Agrobacterium*-mediated co-delivery of the *GRF–GIF* chimeric gene results in the efficient regeneration of transgenic wheat plants

**FIGURE 1:**
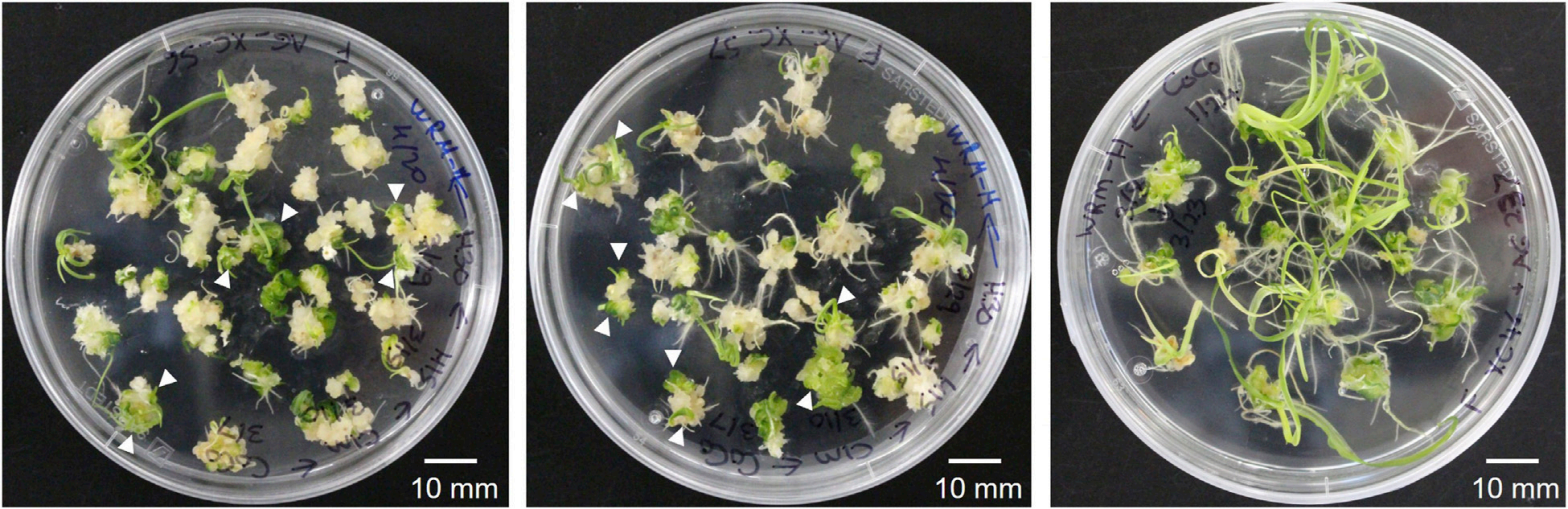
Representative images of the wheat (cv. Fielder) transformation with the *GRF–GIF* chimera using *A. tumefaciens* (AGL1/pSoup). Some calli produce multiple green shoots (shown with white arrowheads), eventually regenerating into individual transgenic seedlings.

The *Agrobacterium*-mediated wheat transformation was done following a recently published protocol (Hayta et al., 2021) with the co-delivery of the wheat transcription factor *GRF4* and its cofactor *GIF1* (Debernardi et al., 2020). The reported transformation efficiency with the improved protocol and the *GRF4–GIF1* construct co-delivery was 77.5% in cv. Fielder (number of transgenic plants per cultured embryos). Similarly, our transformation rate with the JD633 plasmid carrying the *GRF4–GIF1* and Cas9/gRNA expression cassette ranged from 22% to 68% with an average of 44.1% (Table 1, total transformation rate, including both clonal and non-clonal events). Many calli following transfer to the WCI media and later to the WRM generated multiple green shoots (Figure 1) that we treated as single transgenic events. However, we did not exclude the possibility of some of them being clones if they originate from a single callus (not tested in this study). Healthy donor plants and good embryo quality were critical for the efficient regeneration of transgenic plants. It is worth noting that following the co-culturing of embryos on WIM, the embryos are transferred to WCI with timentin to control the *Agrobacterium* overgrowth. The WCI media must be prepared fresh, and the culture plates can be stored in the fridge for up to 2 weeks to prevent timentin from losing its activity.

**TABLE 1:** Typical *Agrobacterium*-mediated transformation rates for wheat (cv. Fielder) with co-delivery of the *GRF–GIF* embryogenesis construct.

Replicate	Cultivar	Agrobacterium strain/helper plasmid	Number of embryos transformed	Number of PCR-confirmed transgenics recovered	Transformation efficiency, %
#1	Fielder	AGL1/pSoup	192	44	22.9
#2	Fielder	AGL1/pSoup	145	70	48.3
#3	Fielder	AGL1/pSoup	190	70	36.8
#4	Fielder	AGL1/pSoup	221	151	68.3
**Average**			187	84	44

### 3.2 Low- and high-copy-number T-DNA integration events can be recovered in the transgenic population

**FIGURE 2:**
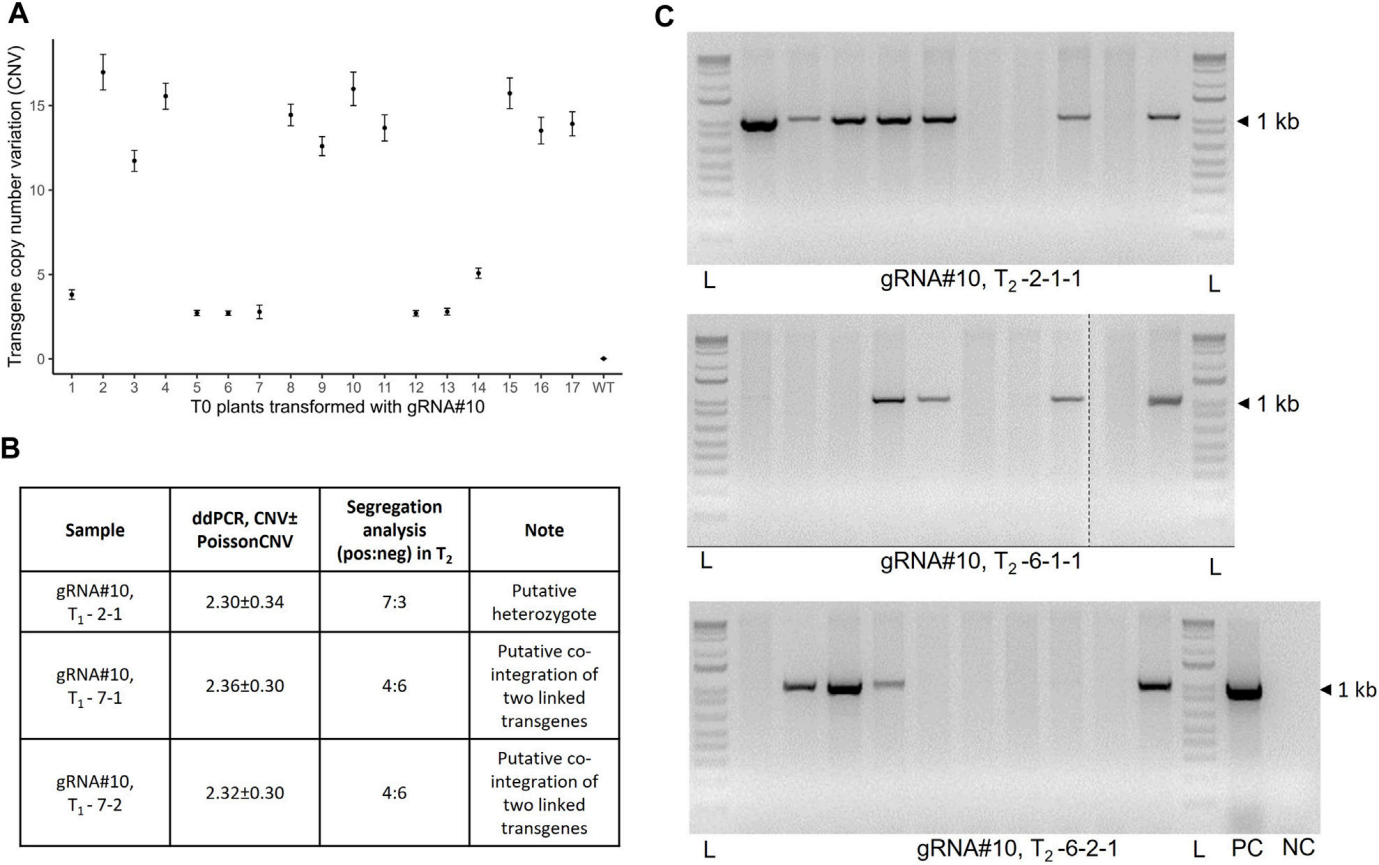
Analysis of the transgene copy number in wheat T_0_ transgenic lines generated using *Agrobacterium*-mediated transformation and their progeny. **(A)** Copy number variation (CNV) of the transgene in the T_0_ plants transformed with JD633::gRNA#10 and estimated using ddPCR. Error bars represent Poisson CNV max and min values. **(B)** Estimation of the CNV values in the selected T_1_ progeny. **(C)** Segregation analysis of the T_2_ population for selected lines (T_2_-2-1-1, T_2_-6-1-1, and T_2_-6-2-1) using hygromycin-specific primers (HYG-F and HYG-R). L, ladder; PC, plasmid positive control; NC, no-template control.

The transgene copy number was measured using the ddPCR assay as described previously (Collier et al., 2017) with some modifications. Based on the wheat genome size (17.33 pg for 1C plant DNA), the original protocol calls for 350 ng of *EcoR*I-digested wheat gDNA/sample to quantify the transgene copy number (Collier et al., 2017). It was also suggested to perform the digestion of gDNA for 12 h. At the same time, another study on the optimization of the *α*-gliadin gene copy number estimation identified the optimal amount to be 10 ng of digested gDNA per 20 µL of ddPCR mixture(Jouanin et al., 2020). Therefore, we compared the T-DNA copy number values between the two different gDNA amounts for 11 T_0_ plants and observed no significant difference in the copy number values between corresponding samples (Supplementary Table S1, Pearson’s correlation R = 0.99, *p* = 1.24e-08). Additionally, a prolonged digestion period (more than 1 h) of the total gDNA before the ddPCR analysis was not required for reproducible estimation of the transgene copy number (Supplementary Table S2, Pearson’s correlation R = 0.97, *p* = 6.98e-08). To our surprise, many T_0_ plants selected for the study (10 out of 17) contained multiple T-DNA copies ranging from 3 to as high as 17 (Figure 2A). These data were further confirmed by estimating the transgene copy number in the T_1_ population and segregation analysis in the T_2_ progeny (Figures 2B, C). Consistent with the recent report (Luo et al., 2021), we did not observe a correlation between the Cas9/gRNA cassette copy number and GE rate in the T_0_ plants and their progeny (Supplementary Table S3, Pearson’s correlation, R = 0.31, *p* = 0.33 and R = 0.24, *p* = 0.27 for T_0_ and T_1_ plants, respectively). At the same time, we observed a moderate but significant positive correlation between the transgene copy number and gene expression in respective samples (Supplementary Figure S1, Pearson’s correlation, R = 0.55, *p* = 0.0049).

### 3.3 Frequency of co-integration events and the integrity of the T-DNA borders

The GE efficiency for the high-throughput functional genomics studies could be increased by co-delivering multiple gRNAs targeting the same gene (Luo et al., 2021). gRNAs could be coded on the single expression plasmid (e.g., tRNA or ribozyme expression cassettes) or the separate plasmids (Abdelrahman et al., 2021). We cloned 10 different seeding sequences into the JD633 plasmid, transformed them separately into *Agrobacterium*, and performed five individual co-transformation experiments by mixing two *Agrobacterium* isolates before the embryo inoculation. We screened 10 T_0_ plants, each regenerated from five independent co-transformation events, with approximately half of them (48% ± 17.8%) being PCR-positive for both gRNAs co-delivered (Supplementary Figure S2).

**FIGURE 3:**
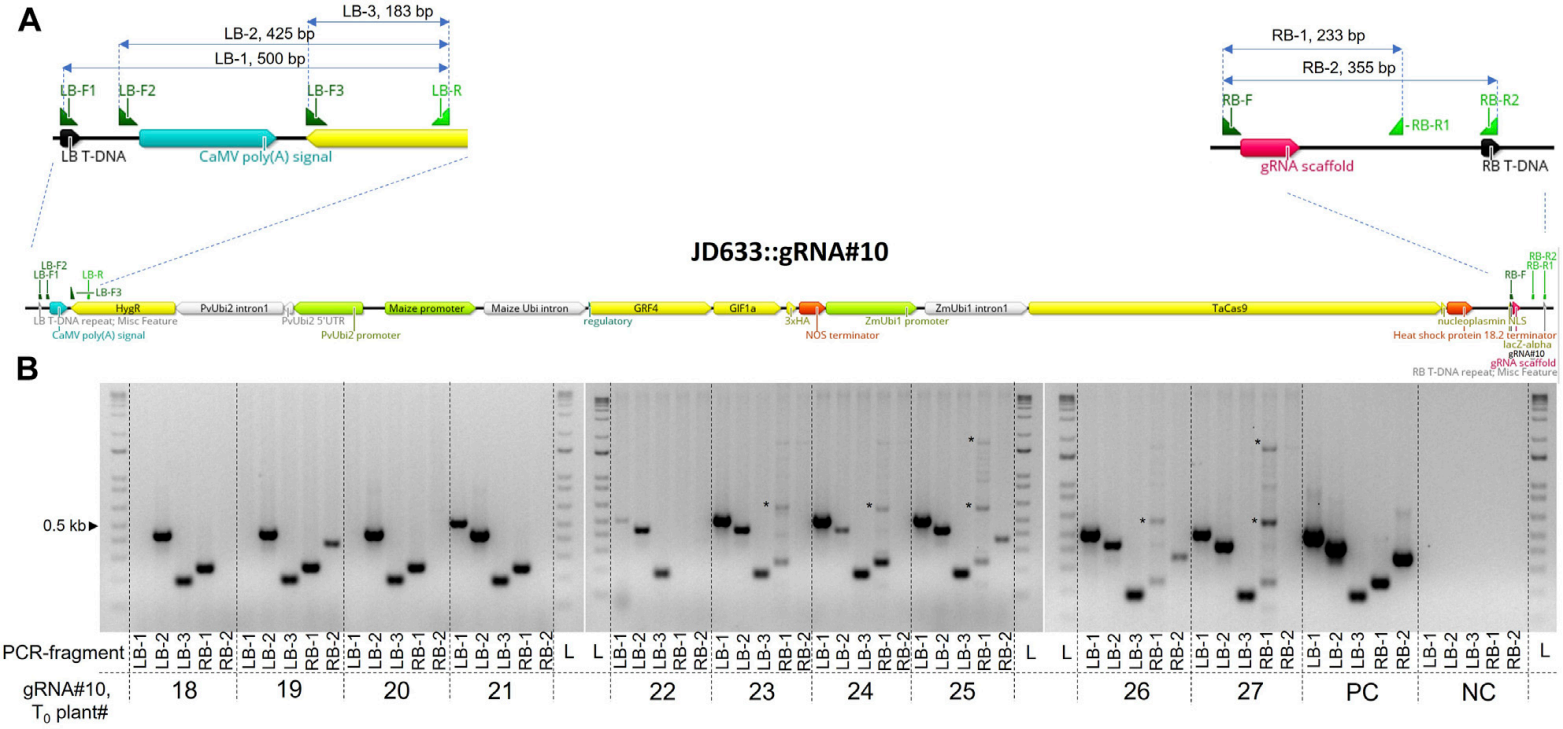
Evaluation of the T-DNA border integrity in T_0_ transgenics generated using *Agrobacterium*-mediated transformation with the JD633::gRNA#10 plasmid. **(A)** Scheme of the JD633::gRNA#10 plasmid with the marked primer-binding sites and PCR fragments generated for the analysis. Primers were designed to anneal either at the left or right borders (LB-F1 or RB-R2) or at different distances from the borders for the PCR-based screening of the T-DNA integrity in transgenic plants. **(B)** Analysis of the PCR fragments on the 1% agarose gel. PC, plasmid positive control; NC, no-template control (cv. Fielder); L, ladder; LB, left border; RB, right border; *, unspecific amplification.

During the *Agrobacterium*-initiated T-DNA delivery, the right border is protected from the exonuclease cleavage by covalently linked VirD2 protein, whereas the left edge is prone to truncations (Gelvin, 2017). For this reason, plant selection cassettes are usually placed closer to the left border to select for transgenic events with intact left and right border regions. We tested the integrity of the left and right T-DNA borders in 10 randomly selected T_0_ transgenic plants transformed with the JD633::gRNA#10 plasmid using primers placed at different distances from the borders. Three separate forward primers (LB-F1, F2, and F3) and one standard reverse primer (LB-R) were used for the left border. For the right border, we used one common forward primer (RB-F) and two reverse (RB-R1 and R2) primers (Figure 3A). Seven out of ten plants tested had an intact left border, whereas only three plants had a complete right border (Figure 3B). At the same time, all but one plant (T_0_ plant #22) had an entire gRNA cassette at the right border, as seen from the presence of the RB-1 PCR fragment. Overall, we observed truncations at both borders. Nevertheless, these deletions were at a distance from the essential components of the cassettes.

### 3.4 *Agrobacterium*-mediated wheat transformation results in the efficient generation of gene-edited plants

Previously, others and we have demonstrated that biolistic-mediated transformation can effectively generate edited wheat plants (Li et al., 2021; Xu et al., 2022). Here, we tested the efficiency of GE with the single-guide RNA (gRNA#10) targeting six copies of the wheat *grain width and weight2* gene (*TaGW2*) (Wang et al., 2018). The gene encodes a RING-type E3 ubiquitin ligase, found initially to regulate rice grain weight by increasing the cell number of spikelet hulls (Song et al., 2007). We analyzed 46 T_0_ lines for the presence of edits at the target sites using the cleaved amplified polymorphic sequence assay (CAPS), with 27 of the lines (59%) demonstrating various levels of edits at three sub-genomes (Supplementary Figure S3). All but one edited line were heterozygous/chimeric for the presence of edits. We randomly selected 12 lines and propagated them into the T_1_ generation to test for the transgenerational inheritance of the edits. The 24 lines (two T_1_s per parental line) were screened for the presence of the transgene cassette and the edits. Fifty percent of the screened progeny contained different levels of edits at the target site, with one plant (T_1_ plant# 9-2, Supplementary Figure S4) having disrupted all six copies of the gene.

**FIGURE 4:**
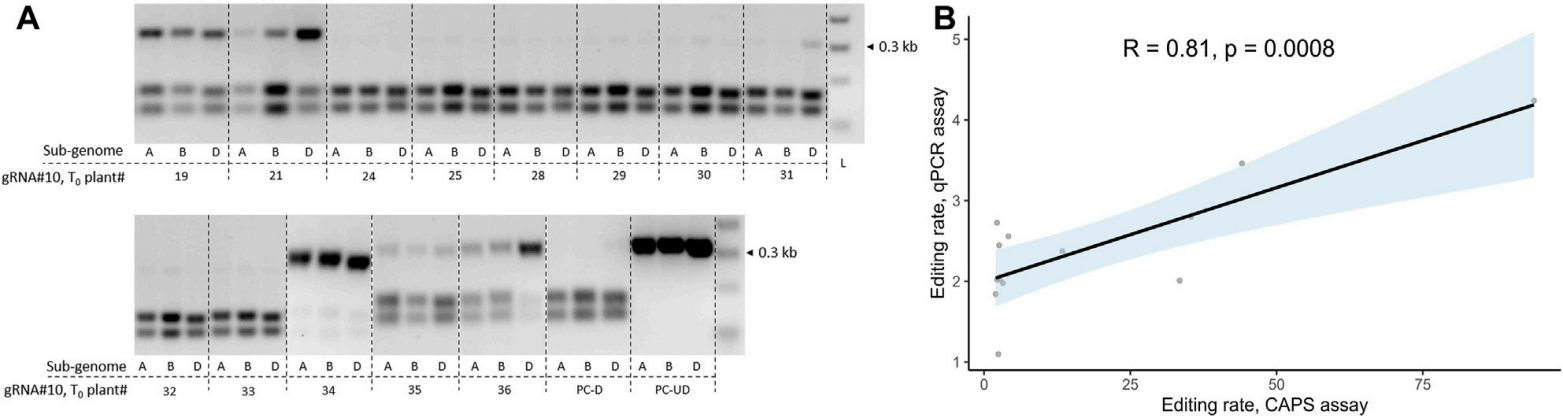
Correlation analysis for GE rate estimation using qPCR or CAPS assay in T_0_ transgenic plants. **(A)** Screening of T_0_ transgenic wheat lines (cv. Fielder) for the presence of GE events at the target loci for three sub-genomes using the cleaved amplified polymorphic sequence (CAPS) assay. The plants were generated using *Agrobacterium*-mediated transformation and co-delivery of the *GRF–GIF* chimeric construct. The gRNA#10 seeding sequence targeting three sub-genomic copies of *TaGW2* was cloned into the JD633 plasmid carrying *GRF–GIF* and Cas9/gRNA expression cassettes. The CAPS assay for three sub-genomes targeted by gRNA#10 was performed as described previously (Xu et al., 2022) using *Sma*I restriction digestion. PC-D, digested WT control (cv. Fielder); PC-UD, undigested WT control (cv. Fielder); L, ladder. **(B)** Correlation analysis between GE rates estimated using the CAPS assay and probe-based qPCR for gRNA#10 in the corresponding T_0_ transgenic plants. The *x*-axis represents the average intensity of the undigested bands for the three sub-genomes of respective T_0_ plants estimated using the CAPS assay. The GE rates for the qPCR assay were calculated through the normalization of ΔΔCt values of transgenic plants to WT control (ΔΔCt_norm_, cv. Fielder), and the 1/ΔΔCt_norm_ ratios were used for plotting. The trendline was plotted with light blue, indicating the standard error. The R- and *p*-values were calculated using Pearson’s correlation coefficient.

Although the CAPS assay remains a highly robust method for detecting the GE events in polyploid crops, it relies on a restriction site at the gRNA cut site, which is not always the case. We decided to compare the GE rate detection between the CAPS assay and the probe-based qPCR method for the T_0_ transgenics generated with JD633::gRNA#10. The qPCR assay is less labor-intensive and allows higher-throughput analysis than the CAPS assay. The GE events were quantified for three sub-genomes in 13 plants for the CAPS assay, and an average GE rate per T_0_ transgenic plant was calculated (Figure 4A). The same 13 plants were analyzed using the qPCR-based method with the universal primers designed to span the target regions for all three sub-genomes with the probe annealing to the gRNA cut site. Eventually, the GE rates for the qPCR assay were calculated through the normalization of ΔΔCt values of transgenic plants to WT control (ΔΔCt_norm_, cv. Fielder), and the 1/ΔΔCt_norm_ ratios were used for plotting. We observed a significant positive correlation between the two quantification assays (Pearson’s correlation, R = 0.81, *p* = 0.0008, Figure 4B). Therefore, we decided to use the qPCR-based assay to quantify GE rates.

**FIGURE 5:**
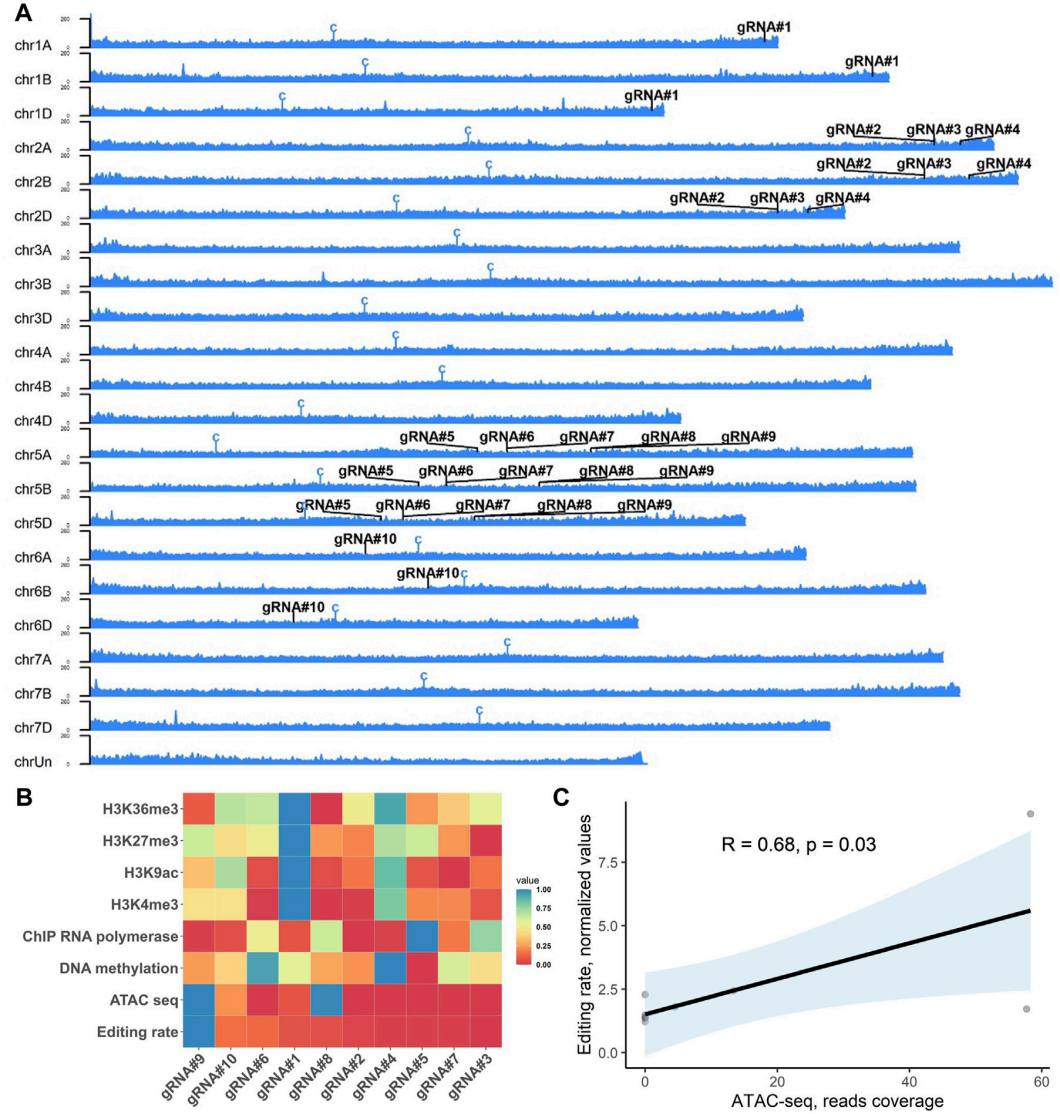
Effect of the epigenetic profile at the target loci on the editing efficiency of selected gRNAs in wheat. **(A)** Ideogram of the ATAC sequencing read distribution along the wheat chromosomes (cv. Chinese Spring, PRJNA552871 (Concia et al., 2020b) with the positions of 10 gRNAs tested in this study. Centromeres are denoted with a “C” symbol. The *Y*-axis scale shows an intensity of mapped reads to the chromosome position. **(B)** Heatmap of the GE rate for individual gRNAs (*x*-axis) for distinct epigenetic marks (*y*-axis). The heatmap represents non-clustered scaled values (from 0 to 1) for the editing rate and nucleotide resolution epigenetic marks at the gRNA target site plus three nucleotides of the PAM sequence (20 + 3 nt, except the gRNA#10 – 19 + 3 nt). **(C)** Correlation analysis of the ATAC-Seq data and GE rates for 10 gRNAs estimated using qPCR. The trendline was plotted with light blue, indicating the standard error. The R- and *p*-values were calculated using Pearson’s correlation coefficient.

We generated 10 constructs with gRNA seeding sequences cloned into the JD633 plasmid, targeting seven genes (Table 2). Every gRNA was designed to mutagenize three gene homeologs per haploid genome with 30 regions (Figure 5A). We generated 65 T_0_ plants with at least three independent plants per gRNA.

**TABLE 2:** gRNAs cloned into the JD633 plasmid and used for *Agrobacterium*-mediated wheat transformation.

gRNA	Target gene	Homeolog ID	Activity score, WheatCrispr (Cram et al., 2019)
gRNA#1	*TRYPTOPHAN SYNTHASE, ALPHA CHAIN* (*TaTRP_SYNTHASE_SUA*)	TraesCS1A02G428400, TraesCS1B02G463100, and TraesCS1D02G437700	0.35
gRNA#2	*INOSITOL TETRA/PENTAPHOSPHATE 2-KINASE *(*TaIPK1*)	TraesCS2A02G497700, TraesCS2B02G525900 and TraesCS2D02G612600LC	0.45
gRNA#3	*INOSITOL TETRA/PENTAPHOSPHATE 2-KINASE* (*TaIPK1*)	TraesCS2A02G497700, TraesCS2B02G525900 and TraesCS2D02G612600LC	0.39
gRNA#4	*MANGANESE SUPEROXIDE DISMUTASE* (*TaMnSOD*)	TraesCS2A02G537100, TraesCS2B02G567600, and TraesCS2D02G538300	0.42
gRNA#5	*WHEAT PROLAMIN-BOX-BINDING FACTOR* (*TaWPBF*)	TraesCS5A02G155900, TraesCS5B02G154100, and TraesCS5D02G161000	0.44
gRNA#6	*DEMETER* (*TaDME*)	TraesCS5A02G169000, TraesCS5B02G165800, and TraesCS5D02G173300	0.46
gRNA#7	*DEMETER* (*TaDME*)	TraesCS5A02G169000, TraesCS5B02G165800, and TraesCS5D02G173300	0.46
gRNA#8	*QUANTITATIVE TRAIT LOCUS ON SEED DORMANCY 1 (TaQSD1)*	TraesCS5A02G216200, TraesCS5B02G214700, and TraesCS5D02G224200	0.47
gRNA#9	*QUANTITATIVE TRAIT LOCUS ON SEED DORMANCY 1 (TaQSD1)*	TraesCS5A02G216200, TraesCS5B02G214700, and TraesCS5D02G224200	0.46
gRNA#10	*GRAIN WIDTH and WEIGHT2* (*TaGW2*)	TraesCS6A02G189300, TraesCS6B02G215300, and TraesCS6D02G176900	0.45

All the italic values in brackets are abbreviations of the genes.

**FIGURE 6:**
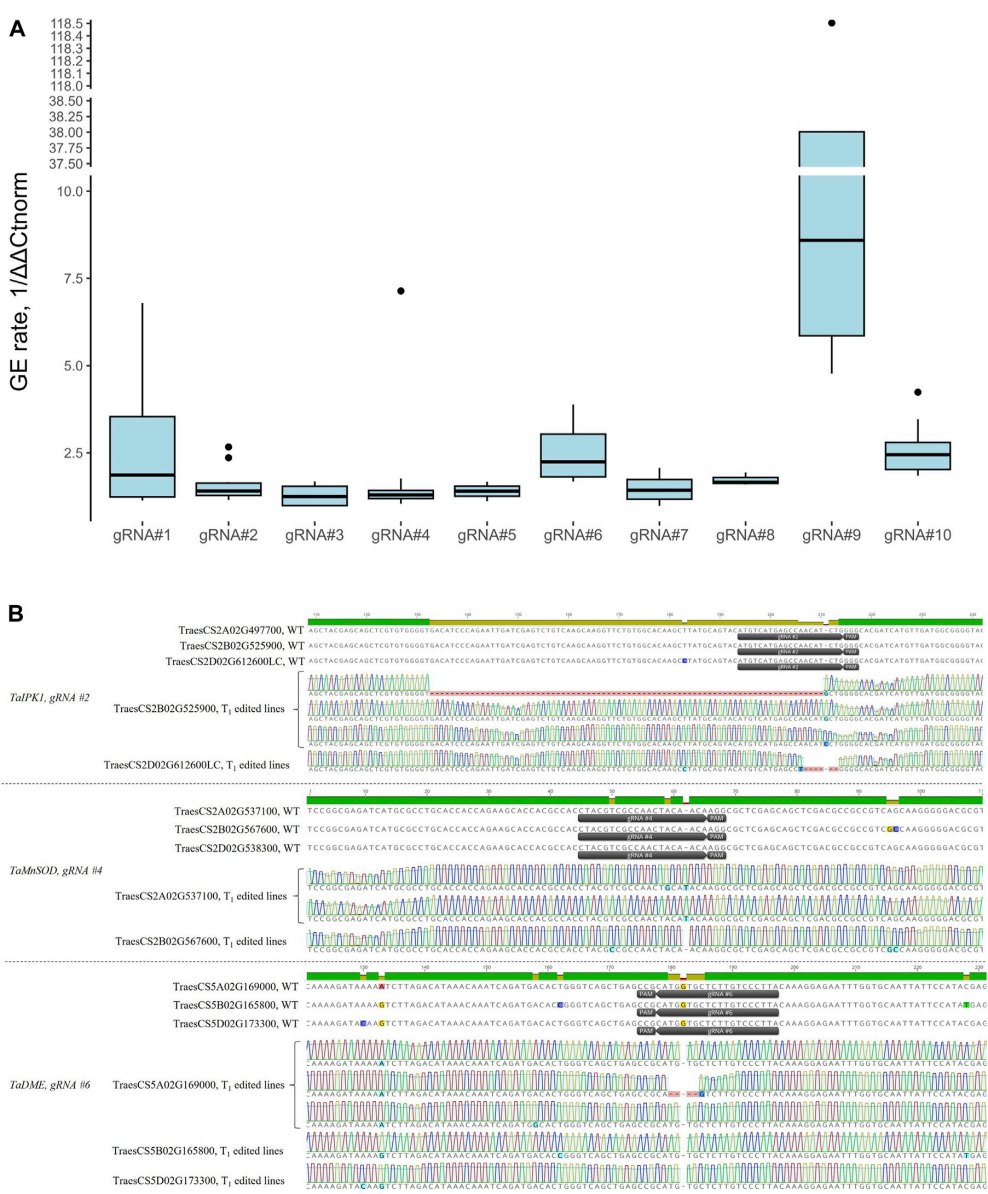
**(A)** Box plots showing relative normalized GE rates for 10 gRNAs in T_0_ transgenic plants quantified using the probe-based qPCR method. At least three independent T_0_ transgenic plants per gRNA were analyzed. The GE rates for the qPCR assay were calculated through the normalization of ΔΔCt values of transgenic plants to WT control (ΔΔCt_norm_, cv. Fielder), and the 1/ΔΔCt_norm_ ratios were used for plotting. **(B)** Representative edits at the target sites as revealed by Primordium sequencing for gRNAs #2, 4, and 6. Target regions were amplified from gDNA of T_1_ plants using either sub-genome specific or universal primers and cloned into the pJET1.2 plasmid for sequencing. Sequence alignment was done using Clustal Omega in Geneious Prime software.

Mean normalized GE rates for T_0_ plants were quantified using the probe-based qPCR method varied from 1.29 to 35.15, with gRNA#9 showing the highest activity (Figure 6A, Supplementary Table S4). We further confirmed the presence of edits at the target site for the gRNAs #2, 4, and 6 using Primordium sequencing (Figure 6B). We detected single-nucleotide insertions, deletions, substitutions, and small and large deletions (from 5 to 78 nucleotides) at the gRNA cut sites. Overall, we observed no correlation between activity scores (Table 2, using models devised by Doench et al., (2016)) and the editing rate of gRNAs tested in our study (Pearson’s R = −0.22, *p* = 0.55).

Editing efficiency in plants can vary depending on gRNA used, with both sequence-dependency and epigenetic profile suggested as the reasons for inconsistent GE rates among different loci (Naim et al., 2020; Weiss et al., 2022). Therefore, we decided to test whether the epigenetic profile at the target loci can affect the mutagenesis rates observed in our study. The nucleotide resolution data for six epigenetic profiles (DNA methylation, H3K4me3, H3K9ac, H3K27me3, H3K36me3, and ATAC-Seq) and RNA polymerase II-ChIP (Concia et al., 2020a; Jia et al., 2021) for corresponding gRNAs were extracted from available datasets for wheat seedlings (cv. Chinese Spring) and correlated with the mean GE rates observed in Figure 6A. We observed a significant positive correlation between the average mutagenesis rates for 10 gRNAs and ATAC-Seq data (Pearson’s correlation, R = 0.68, *p* = 0.03, Figures 5B, C). At the same time, no correlation was observed for the rest of the epigenetic marks analyzed (Supplementary Table S5).

## 4 Discussion

Here, we characterized transgenic and GE events in wheat generated with the co-delivery of the *GRF4–GIF1* using *Agrobacterium*-mediated transformation (Hayta et al., 2021). The method is based on a high-efficiency proprietary transformation protocol (PureWheat^®^ technology) developed by Japan Tobacco Inc. (Shizuoka, Japan) for the wheat cv. Fielder (Richardson et al., 2014; Ishida et al., 2015). The protocol has been successfully applied to transform Chinese and Australian commercial varieties (Richardson et al., 2014; Wang et al., 2017) with up to 50% transformation efficiencies. In our hands, the expression of a *GRF4–GIF1* chimera (Debernardi et al., 2020) was required to achieve high transformation efficiency from 22% to 68% of the cv. Fielder. This is a significant increase from the biolistic-mediated transformation (0.1%–1%) previously used in our study (Xu et al., 2022). Similarly to the recently developed “QuickWheat” transformation method with overexpression of maize *WUS* and *BBM* genes (Johnson et al., 2023), we observed a low 5% escape rate on hygromycin selection, as confirmed by PCR. The quality of transgenic events was evaluated by estimating the transgene copy number and integrity of the T-DNA borders. Most T_0_ plants tested had multicopy transgene integrations and sequence truncations at the right and left borders. At the same time, low-copy-number (two and three copies) transgenic events could still be generated and were faithfully propagated into the following generation. Similarly, the border truncations were limited only to the borders of the T-DNA region and did not affect the essential components of the transgene. High-copy-number transgene integration events can lead to transgenerational transgene silencing (Gelvin, 2003) and complicate screening for transgene-free plants with the presence of edits. It needs to be further explored if the quality of transgene events can be controlled by manipulating the transformation conditions or using alternative embryogenesis genes. Additionally, it will be essential to test for the presence of the sequences outside of the T-DNA region (plasmid backbone) in transgenic plants to calculate the rate of the “quality event (QE),” as shown in other crops (Che et al., 2022).

Overcoming the bottleneck of stable wheat transformation opens the possibility for population-scale genetic engineering using the CRISPR/Cas9 technology. Targeted mutagenesis at the population scale allows for rapid hypothesis-driven screening of the candidate genes with CRISPR/Cas9-generated multiple alleles for the presence of a desirable phenotype. Such an approach has been successfully implemented in rice, where high transformation rates allow for efficient generation of transgenic population with diverse gRNAs. For example, generating a large-scale mutant library was possible by targeting 12,802 genes highly expressed in rice shoot base tissue with more than 25,000 corresponding sgRNAs (Meng et al., 2017).

Co-delivery of multiple gRNA constructs for population-scale gene editing can result in the co-integration events per single plant rather than generation of separate plants with unique gRNA insertions. On average, we observed 48% ± 17.8% co-integration events in five independent co-transformation experiments. It remains to be shown whether co-transformation with the higher number of gRNAs could lead to the transgenic population with fewer co-integration events/plants. The ability to generate a high proportion of plants with the single-gRNA integrations from the embryos infected with the *Agrobacterium* mixture allows for the high-throughput-targeted mutagenesis of the coding sequences or regulatory regions of the genes with the following phenotyping of the regenerated edited population. On the other hand, if the mutations act synergistically, a combination of a few gRNAs targeting different genes in a single plant could be beneficial for the fast recovery of the mutants with a desired phenotype. We successfully recovered T_0_ plants with GE events at three sub-genomes as determined using CAPS and probe-based qPCR assays (Figure 4, Supplementary Figure S3) for the previously tested gRNA#10 (Xu et al., 2022). The mutagenized loci were faithfully propagated into the following generation (Supplementary Figure S4). We further evaluated the GE rate for nine more gRNAs targeting six genes or 27 regions across the wheat genome (sub-genomes A, B and D, Figure 5A). We observed a drastic difference in the mutagenesis efficiency among tested gRNA (over 27-fold difference between the least and the most active gRNAs, Figure 6) with no correlation between GE rate and activity scores using models devised by Doench et al. (2016) (Pearson’s R = −0.22, *p* = 0.55).

Recent research has shown that the chromatin contexts play a crucial role in the effectiveness of CRISPR/Cas9-mediated genome editing, as highlighted in several studies (Wu et al., 2014; Daer et al., 2017; Yarrington et al., 2018; Liu et al., 2019). These investigations have consistently revealed that the presence of heterochromatic features within the CRISPR target regions can hinder the efficiency of CRISPR/Cas9 mutagenesis in various systems, including yeast, *Arabidopsis*, rice, mouse, and human cell lines (Wu et al., 2014; Daer et al., 2017; Yarrington et al., 2018; Liu et al., 2019; Weiss et al., 2022). Similar to the previous studies (Weiss et al., 2022), we show that the gRNA activity had a significant positive correlation with the epigenetic profile and chromatin accessibility as revealed by ATAC-Seq data for leaves in 14-day-old seedlings (Concia et al., 2020a) (Pearson’s correlation, R = 0.68, *p* = 0.03, Figure 5C). At the same time, there was no relationship between other epigenetic signatures (DNA methylation, H3K4me3, H3K9ac, H3K27me3, H3K36me3, and RNA polymerase II-ChIP) and mutagenesis rate for 10 gRNAs in our experiments (Figure 5A and Supplementary Table S5). The JD633 plasmid used in our study contains a constitutive maize Ubi promoter that drives the Cas9 expression, and the gRNA expression is driven by the TaU6 promoter. If Cas9 and gRNA are actively expressed in calli, targeted mutations could accumulate early in plant regeneration. In the future, it will be essential to examine Cas9 protein expression through the Western blot at the different stages of calli development. This information can aid in determination of the most appropriate stage for the evolution of the epigenetic profile in calli produced using *GRF–GIF* co-delivery and correlate it to GE rates at different loci to conclude the contribution of these factors to the CRISPR/Cas9 mutagenesis. In the current study, we used ChIP-Seq, DNA methylation, and ATAC-Seq data generated from the leaves of wheat seedlings that may differ from the epigenetic profile in developing calli due to somaclonal variation (Miguel and Marum, 2011; Lee and Seo, 2018). Moreover, *GRF–GIF*-induced embryogenesis may lead to more accessible chromatin necessary for the highly active transcription in dividing cells. Overall, careful epigenetic profiling of the developing calli induced with *GRF–GIF* expression may provide additional information on the role of chromatin marks in CRISPR/Cas9 mutagenesis.

## Data Availability

The datasets presented in this study can be found in online repositories. The names of the repository/repositories and accession number(s) can be found in the article/Supplementary Material.
