# Correction: Diversity of transgene integration and gene-editing events in wheat (*Triticum aestivum* L.) transgenic plants generated using *Agrobacterium*-mediated transformation

**DOI:** 10.3389/fgeed.2026.1787940

**Published:** 2026-02-19

**Authors:** Louie Cris Lopos, Natalia V. Bykova, Janeen Robinson, Susan Brown, Kerry Ward, Andriy Bilichak

**Affiliations:** Agriculture and Agri-Food Canada (AAFC), Morden Research and Development Centre, Morden, MB, Canada

**Keywords:** wheat, *Triticum aestivum* L., *Agrobacterium*-mediated transformation, gene editing, CRISPR/Cas9, epigenetics

In the published article, an error was identified in the Methods section regarding the species name of *Agrobacterium* used for wheat transformation. The organism was incorrectly listed as *Arabidopsis tumefaciens*.

A correction has been made to section 2 Materials and methods, 2.2 Agrobacterium-mediated wheat transformation, Paragraph Number 1. The correct statement is as follows: “The transformation was performed using the hypervirulent *Agrobacterium tumefaciens* strain AGL1 carrying the pSoup helper plasmid and the JD633 plasmid containing the cloned gRNA seed sequences.”

The original article has been updated.

