# Supplementary material for: Diversity of transgene integration and gene-editing events in wheat (Triticum aestivum L.) transgenic plants generated using Agrobacterium-mediated transformation
Source: Front Genome Ed. 2023 Dec 19;5:1265103. doi: 10.3389/fgeed.2023.1265103 (PMC10773716; doi:10.3389/fgeed.2023.1265103)
Supplement: Supplementary file 1 [file Presentation1.PPTX]

## Slide 1
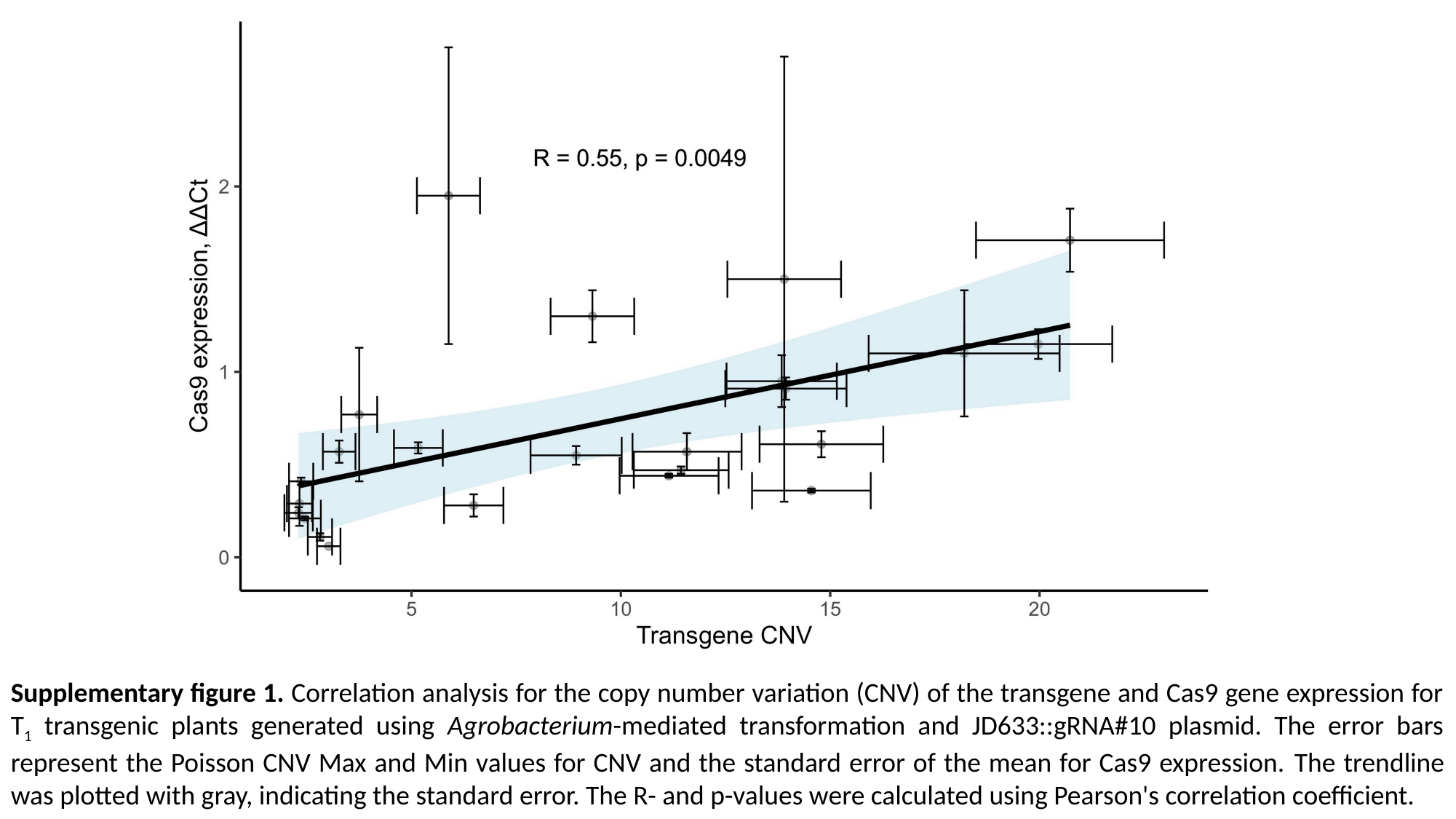

Supplementary figure 1. Correlation analysis for the copy number variation (CNV) of the transgene and Cas9 gene expression for T1 transgenic plants generated using Agrobacterium-mediated transformation and JD633::gRNA#10 plasmid. The error bars represent the Poisson CNV Max and Min values for CNV and the standard error of the mean for Cas9 expression. The trendline was plotted with gray, indicating the standard error. The R- and p-values were calculated using Pearson's correlation coefficient.

## Slide 2
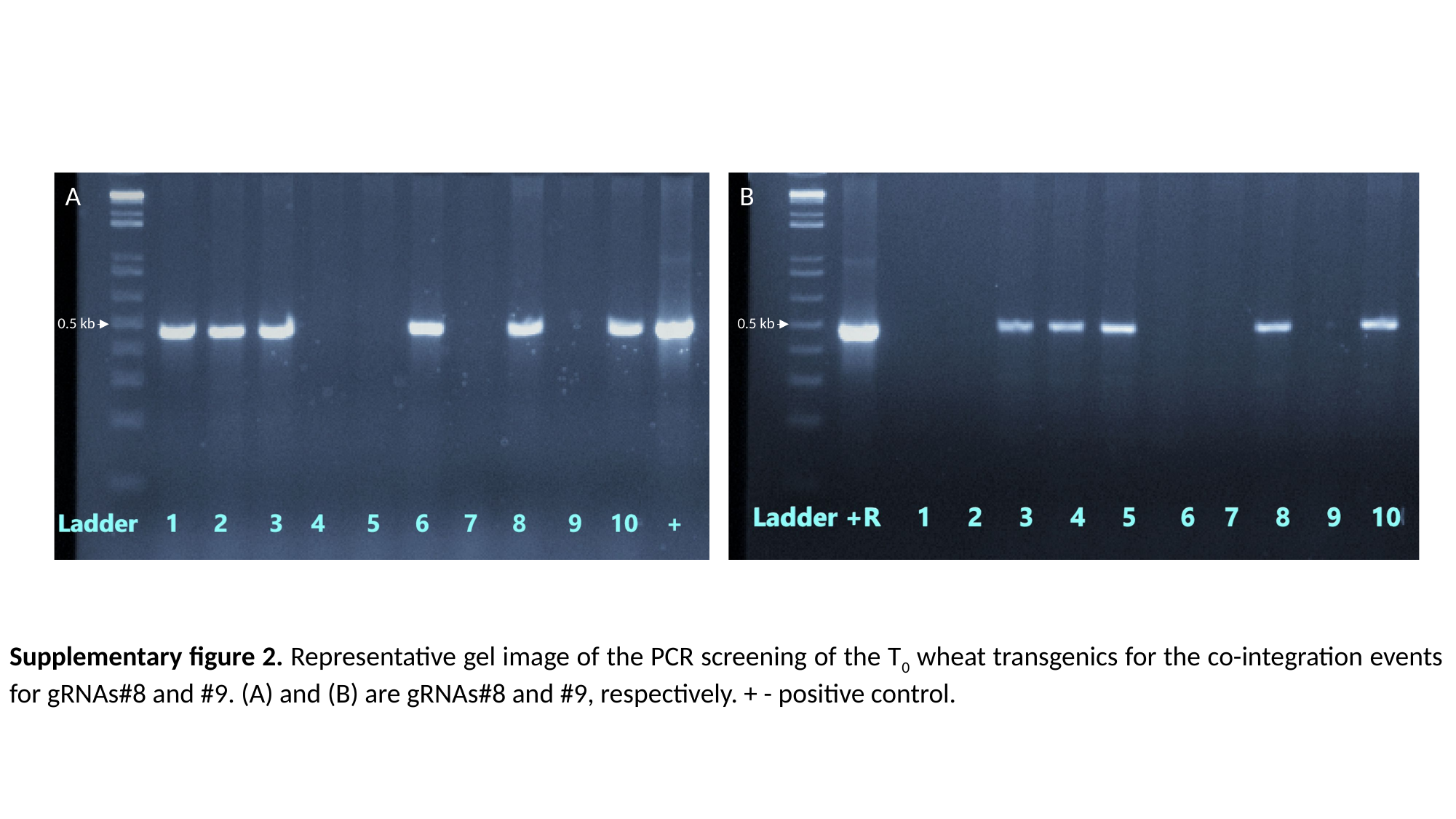

A
B
0.5 kb
0.5 kb
Supplementary figure 2. Representative gel image of the PCR screening of the T0 wheat transgenics for the co-integration events for gRNAs#8 and #9. (A) and (B) are gRNAs#8 and #9, respectively. + - positive control.

## Slide 3
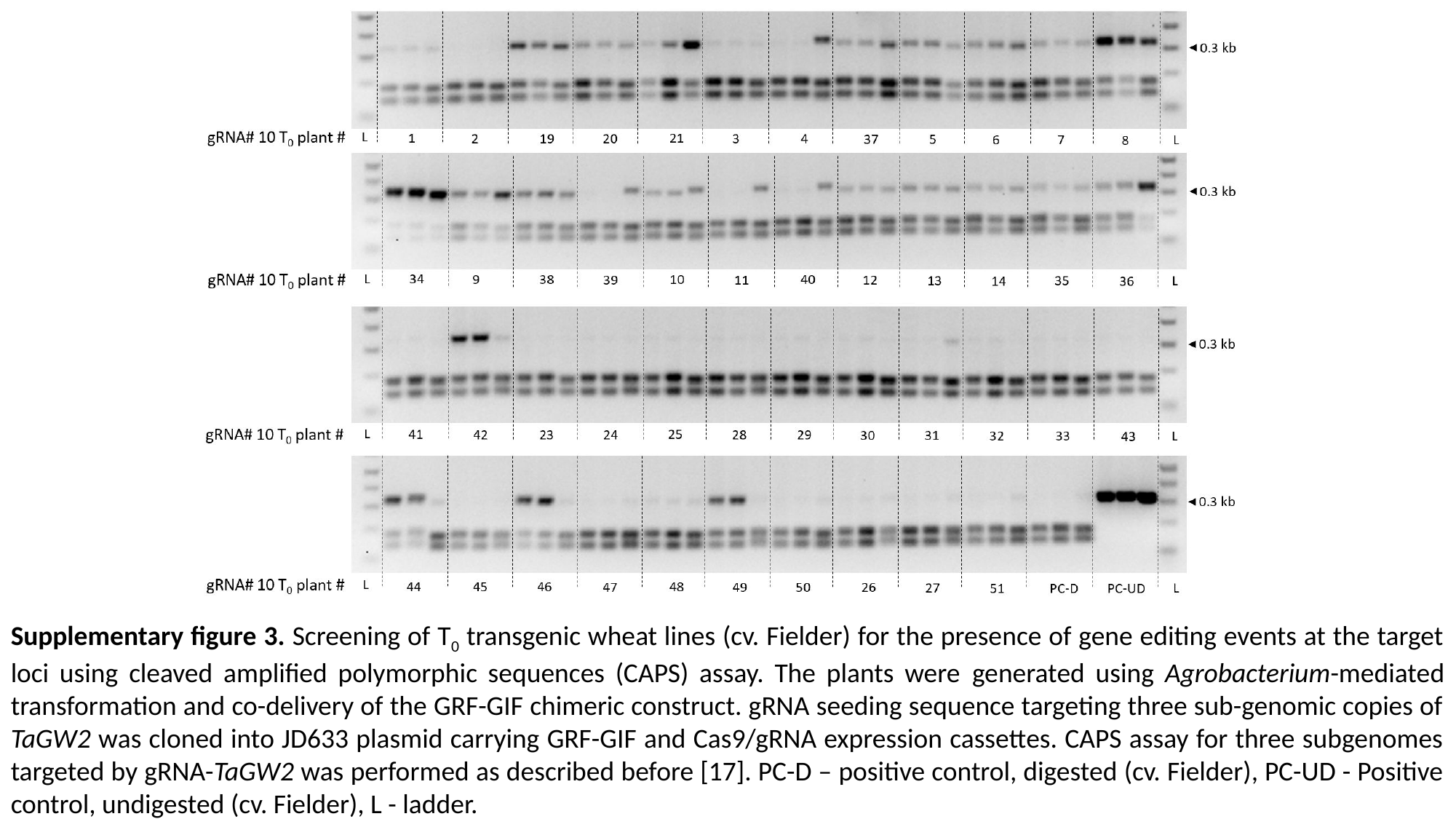

Supplementary figure 3. Screening of T0 transgenic wheat lines (cv. Fielder) for the presence of gene editing events at the target loci using cleaved amplified polymorphic sequences (CAPS) assay. The plants were generated using Agrobacterium-mediated transformation and co-delivery of the GRF-GIF chimeric construct. gRNA seeding sequence targeting three sub-genomic copies of TaGW2 was cloned into JD633 plasmid carrying GRF-GIF and Cas9/gRNA expression cassettes. CAPS assay for three subgenomes targeted by gRNA-TaGW2 was performed as described before [17]. PC-D – positive control, digested (cv. Fielder), PC-UD - Positive control, undigested (cv. Fielder), L - ladder.

## Slide 4
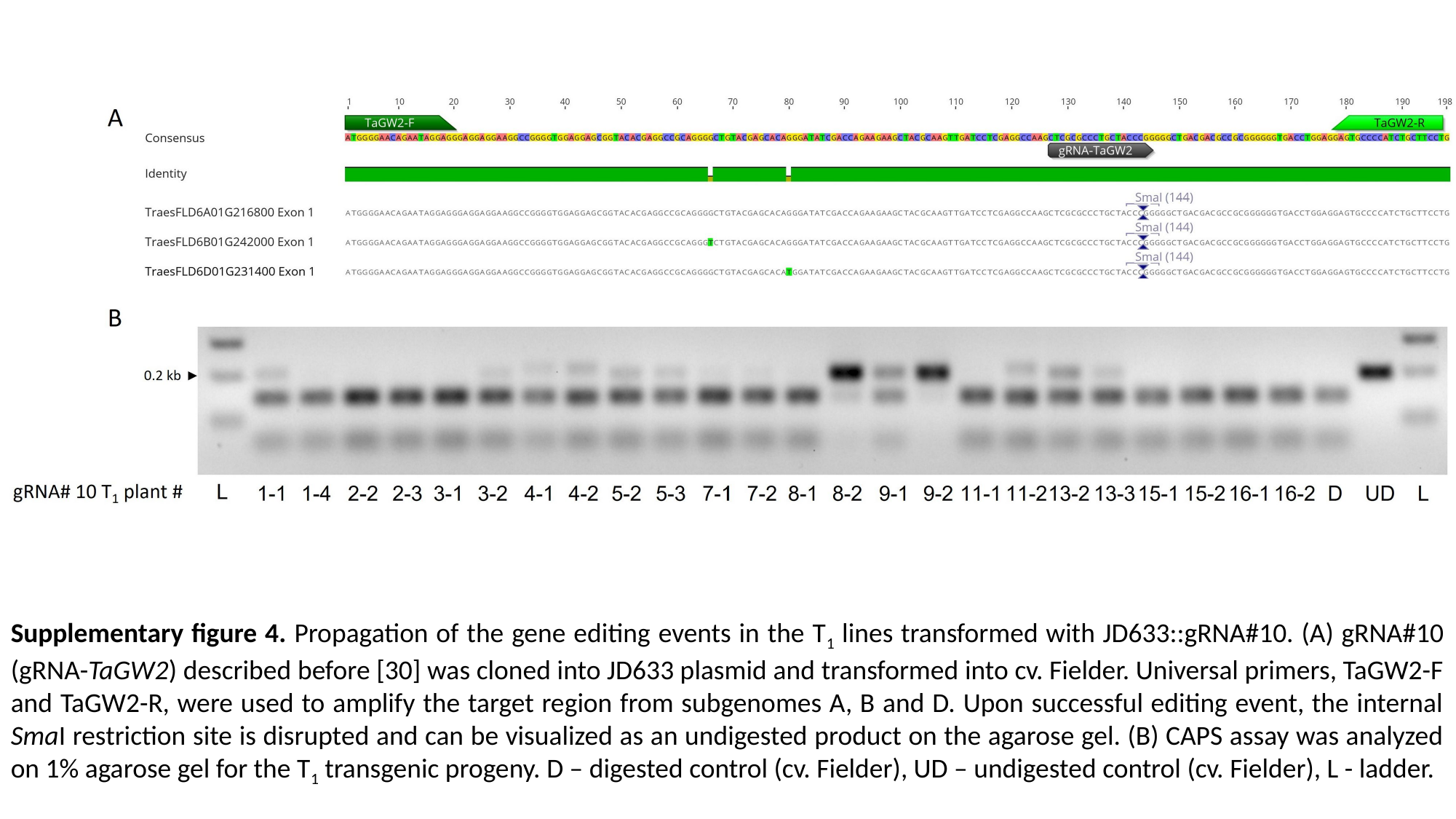

Supplementary figure 4. Propagation of the gene editing events in the T1 lines transformed with JD633::gRNA#10. (A) gRNA#10 (gRNA-TaGW2) described before [30] was cloned into JD633 plasmid and transformed into cv. Fielder. Universal primers, TaGW2-F and TaGW2-R, were used to amplify the target region from subgenomes A, B and D. Upon successful editing event, the internal SmaI restriction site is disrupted and can be visualized as an undigested product on the agarose gel. (B) CAPS assay was analyzed on 1% agarose gel for the T1 transgenic progeny. D – digested control (cv. Fielder), UD – undigested control (cv. Fielder), L - ladder.
